# Combining Electrochemical Impedance Spectroscopy and Surface Plasmon Resonance into one Simultaneous Read-Out System for the Detection of Surface Interactions

**DOI:** 10.3390/s131114650

**Published:** 2013-10-29

**Authors:** Thijs Vandenryt, Andrea Pohl, Bart van Grinsven, Ronald Thoelen, Ward De Ceuninck, Patrick Wagner, Jörg Opitz

**Affiliations:** 1 Institute for Materials Research IMO, Hasselt University, Wetenschapspark 1, 3590 Diepenbeek, Belgium; E-Mails: bart.vangrinsven@uhasselt.be (B.G); ronald.thoelen@xios.be (R.T.); ward.deceuninck@uhasselt.be (W.C); patrick.wagner@uhasselt.be (P.W.); 2 XIOS University College, Agoralaan building H, 3590 Diepenbeek, Belgium; 3 Fraunhofer Institute for Non Destructive Testing (IZFP), Maria-Reiche-Strasse 2, 01109 Dresden, Germany; E-Mails: andrea.pohl@izfp-d.fraunhofer.de (A.P.); joerg.opitz@izfp-d.fraunhofer.de (J.O.); 4 IMEC vzw, IMOMEC, Hasselt University, Wetenschapspark 1, B-3590 Diepenbeek, Belgium

**Keywords:** SPR, EIS, flow-cell, lab on chip, Protein A, immunoglobulin

## Abstract

In this article we describe the integration of impedance spectroscopy (EIS) and surface plasmon resonance (SPR) into one surface analytic device. A polydimethylsiloxane (PDMS) flow cell is created, matching the dimensions of a commercially available sensor chip used for SPR measurements. This flow cell allowed simultaneous measurements between an EIS and a SPR setup. After a successful integration, a proof of principle study was conducted to investigate any signs of interference between the two systems during a measurement. The flow cell was rinsed with 10 mM Tris-HCl and 1× PBS buffer in an alternating manner, while impedance and shifts of the resonance angle were monitored. After achieving a successful proof of principle, a usability test was conducted. It was assessed whether simultaneous detection occurred when: (i) Protein A is adsorbed to the gold surface of the chip; (ii) The non-occupied zone is blocked with BSA molecules and (iii) IgG1 is bound to the Protein A. The results indicate a successful merge between SPR and EIS.

## 1. Introduction

Since the early development of biosensors [1], biosensor technology has successfully been used in many applications and has become more important in recent years for the detection of various analytes. Within biosensor technology surface plasmon resonance (SPR) is considered to be a real-time and label-free tool [2]. Over the past two decades SPR has found its way into medical diagnostics [3–5], environmental analysis [6–9] and food control [9–12]. The working principle is based on changes in the refractive index at interfaces. In the case of biosensors these changes are associated with the binding of the molecules of interest onto a functionalized sensor surface. Though SPR is considered to be a valuable tool, its functionality in some applications might be improved through combined technologies [13–15]. SPR can only measure a limited height above the sensor surface, usually 200 nm or less, for the device used in this paper [16,17], and does not provide any spatial information regarding the binding of a target molecule to its receptor. As an example, we have shown recently that a combination of impedance spectroscopy (EIS) with confocal laser scanning microscopy (CLSM) will lead to a better understanding of the binding kinetics of DNA [18]. Impedance spectroscopy has already been used successfully for monitoring DNA hybridization and the detection of single nucleotide polymorphisms [19], recognition of small molecules by means of molecular imprinted polymers [20] and the detection of C-reactive protein (CRP) [21]. The impedance-based detection offers the possibility of device miniaturization and an electronic read-out. Besides these advantages, EIS does not have the 200 nm limitation and even allows tuning of the penetration depth [15,22]. To investigate the power of a combined SPR and EIS measurement, a PDMS flow-through cell was developed to integrate with the dimensions of the disposable sensor chips, normally used for SPR measurements. First it was studied whether or not the systems would interfere with each other, when simply alternating buffer-media. After proving that the systems were not influencing each other, sensor chips were functionalized with Protein A as receptor for immunoglobulin-G (IgG1) [23]. The binding of IgG1 was then monitored simultaneously by SPR and EIS.

## 2. Experimental

### 2.1. Surface Plasmon Resonance

**Figure 1.**
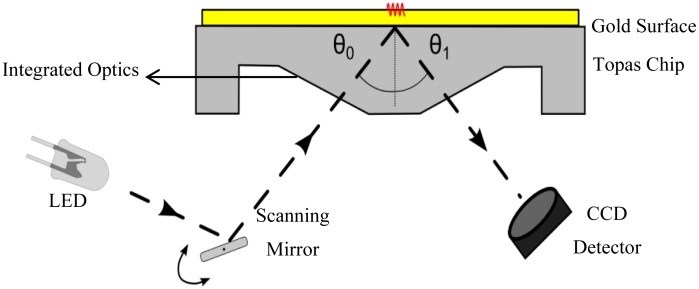
Shows the operating principle of the capitalis SPR device: Light from a LED array is projected on the gold surface, through a scanning mirror and optical elements. A CCD detector is used to detect the intensity of the reflected light. A computer is used to detect and track the minimum, associated with a specific angle of incidence.

The optical phenomenon of SPR occurs on the interface between a metal surface and a dielectric. When light strikes the surface of a certain metal, there is a minimum of the reflected light intensity. The angle of this minimum (resonance angle, θ) is a property of the plasmon resonance and depends on the refractive index of the medium and the wavelength used. The adsorption of molecules to this interface results in a change of the refractive index, which can be detected as a shift of the resonance angle. The change in angle is reported as resonance units (RU): one unit corresponds with the binding of 1 pg of analyte per mm^2^; an angle change of roughly 0.1° corresponds to 1,000 RU. The SPR measurements in this study were performed using the SPR measurement system, developed by Fraunhofer and sold by capitalis technology GmbH (Berlin, Germany). The system consisted of an SPR-read-out unit, combined with an automated on-chip microfluidic system. For the SPR measurements, commercially available, disposable, 76 × 26 × 4 mm^3^ (equal in area to a standard microscopy slide) sensor chips were used, that were molded from a cyclic olefin copolymer referred to as Topas® (KDS Radeberger Präzisions-Formen-und Werkzeugbau, Radeberg, Germany). An area of 12 × 3 mm^2^ on the chip surface was covered with a 50 nm thick gold layer, serving as the metal interface onto which antibodies are grafted. The angle of incidence of collimated light from light emitting diodes (LEDs with a wavelength of 810 nm) was scanned over the underside of the gold area of the chip, through a scanning mirror. The reflected light was detected by a high resolution CCD camera. This is schematically represented in Figure 1. A CPU controlled syringe pump (MLE, Dresden, Germany) was used to administer fluids with programmable flow rates, ranging from 1.25 to 689 μL/s.

### 2.2. Electrochemical Impedance Spectroscopy

Electrical impedance is the measure of the complex resistance which is experienced by a current when a voltage is applied. Using a direct current (DC) can result in deterioration of the electrodes due to corrosion or even destruction of a sensor surface because of hydrolysis. In order to do the biological measurements, it is necessary to perform electronic measurements based on alternating current at an offset voltage lower than 100 mV. Electrochemical Impedance Spectroscopy has an additional advantage; the resulting data can be fitted to an equivalent circuit [24]. This makes it possible to investigate the contribution of different components to the total impedance. The impedance spectroscopy unit, used in this study is a homemade device, earlier described in [24]. This unit measures the impedance in a frequency range of 100 Hz to 100 kHz built up logarithmically with 10 frequencies per decade and a scanning speed of 5.7 s per sweep. All data discussed below refer to a frequency of 251 Hz, ensuring an optimal signal-to-noise ratio. The peak-to-peak amplitude of the AC voltage was limited to 10 mV.

### 2.3. Combining SPR and EIS

**Figure 2.**
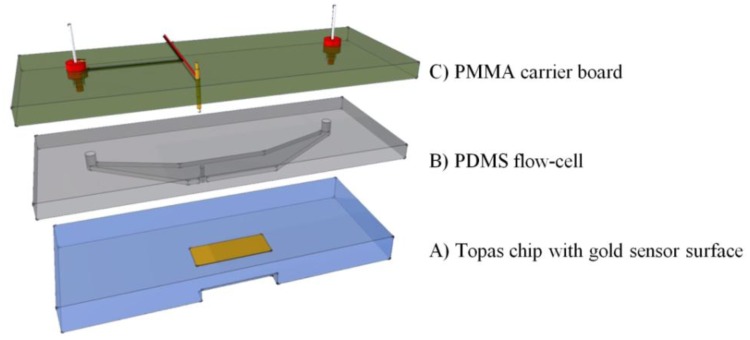
(**A**) The Topas chip with gold sensor surface is covered with a PDMS flow-cell (**B**). The reaction-chamber of the flow-cell is positioned directly over the gold patch of the Topas chip. The flow-cell is permanently bound to a transparent PMMA (Poly methyl methacrylate) carrier board (**C**), carrying the necessary electrical and fluidic connections.

To combine the surface plasmon resonance setup with the impedance spectroscopy unit, a flow-cell was developed. This cell was designed to match the dimensions of the existing Topas® chip. The dimensions are 76 × 26 × 4 mm^3^, a patch of gold (12 × 3 mm^2^, with a thickness of 50 nm) is deposited on top of the slide. The chip is shown in Figure 2A. A PDMS (polydimethylsiloxane) flow-cell is key to achieve a successful combination between the two technologies. PDMS is a transparent flexible silicone elastomer, it is biocompatible and has a high degree of chemical inertness. Its reusability and self-sealing properties make it an ideal material for this application. Figure 2B shows the PDMS flow-cell. To fabricate this cell, a mold is fabricated in PTFE (polytetrafluoroethylene/Teflon; PCH Technischer Handel, Dresden, Germany) to serve as the master template, through computer numerical control (CNC) milling [25]. This master mold is then encapsulated in the PDMS base polymer (Sylgard 184, Dow Corning, Midland, MI, USA), which is mixed thoroughly in a 10:1 ratio with its curing agent. The mixture is degassed for 30 min at an absolute pressure of 50 kPa to remove trapped gas bubbles. Complete curing of the PDMS in an oven at 65 °C takes approximately 3 h. The cured daughter-mold can be gently peeled from the master template and cut to the required dimensions with a lancet. Cavities for the inlet, outlet and spring contact are present in the master mold, so no post-treatment to the PDMS mold is necessary. The resulting reaction-chamber of the flow-cell is positioned directly over the gold patch. A small part of the gold sensor surface is sacrificed to allow a gold plated spring contact to make a connection without any contact with the fluid compartment. The spring contact is pressed gently onto the gold surface of the Topas® chip, ensuring a good electrical connection. As the second connection, a gold wire with a diameter of 500 μm is placed in the outlet compartment of the flow-cell, closing the electrical circuit from the spring contact, to the gold plated patch, through the reagents, to the counter-electrode and finally back to the impedance analyzer. The PDMS flow-cell is permanently bound to a transparent polymethylmethacrylate (PMMA) carrier board. This board acts as a strain relief for the electrical connections and has integrated HPLC (high performance liquid chromatography) fittings to simplify the fluidic handling, as shown in Figure 2C.

The volume of the entire flow-cell is 17.6 μL, with a height of 150 μm. The PMMA and PDMS assembly has a combined height of about 6 mm (2 mm PDMS slab). This mimics the height of the original capitalis flow-cell and ensures sufficient pressure to seal the flow-cell in the snap-fitted SPR unit.

### 2.4. Proof of Principle

To assess the working principle of the combined unit, the reaction chamber of the PDMS flow-cell was filled sequentially with 10 mM Tris-HCl (Carl Roth, Karlsruhe, Germany) and 1× PBS buffer (pH 7.4; GIBCO Life Technologies GmbH, Darmstadt, Germany). These analytes were chosen because they can be distinguished clearly both by impedance spectroscopy and surface plasmon resonance. First 500 μL of 10 mM Tris-HCl is pumped into the reaction chamber. Impedance is measured in a frequency range of 100 Hz–100 kHz. At the same time a surface plasmon resonance measurement is performed. After obtaining a stable signal for both techniques, the medium is exchanged with 500 μL of 1× PBS buffer. This process is repeated three times to verify that the initial stable values for both techniques are reproducible and stay constant in time.

### 2.5. Usability Test

**Figure 3.**
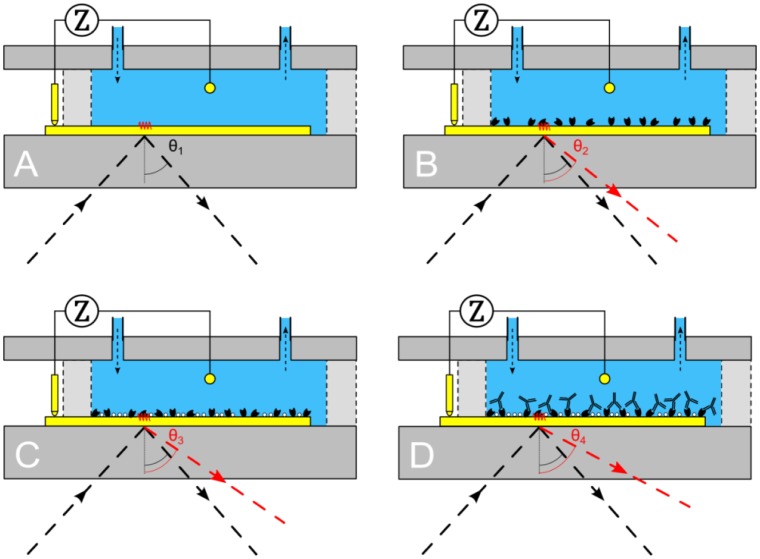
Different stages throughout the usability test. (**A**) Flow-cell is rinsed with 10 mM Tris-HCl solution, nothing is bound to the sensor surface. (**B**) Protein A is attached through physical adsorption to the gold surface, acting as a receptor for IgG1. (**C**) Non-occupied zones of the sensor surface are blocked with BSA molecules. (**D**) IgG1 is bound to the Protein A.

After achieving a successful proof of principle a usability test was conducted. It was studied whether the binding of IgG1 (Sigma-Aldrich Biochemie GmbH, Taufkirchen, Germany) to Protein A (biopur, Reinach, Switzerland) could be observed, both by EIS and SPR in real time. First, the gold patch of the Topas® chip is cleansed by means of a 65% HNO_3_ solution then it is neutralized in a solution containing 25% NH_3_, 30% H_2_O_2_ (all three purchased at Carl Roth) and ultrapure water (Direct-Q3 UV system—Millipore, Schwalbag, Germany) in a ratio of 1:1:5 for 2 min. Subsequent rinsing with ultrapure water and drying under N_2_ atmosphere finalized the cleaning procedure. After this cleaning procedure, the Topas® chip, PDMS flow-cell and PMMA carrier board are assembled. First, the reaction chamber was filled with 500 μL of 10 mM Tris-HCl solution (Figure 3A), to obtain a stable signal for both EIS and SPR. Next, 500 μL of 0.25 mg/mL Protein A solution was added and left for 90 min to be adsorbed to the gold surface (Figure 3B). In the next step, the gold surface was rinsed with 500 μL of 10 mM Tris-HCl solution, to remove the unbound Protein A. When a stable signal was obtained, 500 μL of 1 mg/mL BSA solution was added for 20 min, to block the unoccupied areas of the gold surface (Figure 3C). Then, the system was rinsed again with 500 μL of 10 mM Tris-HCl solution. Upon observing a stable signal, 500 μL of 0.565 mg/mL IgG1 solution was added to the reaction chamber, to bind with the Protein A, for 90 min. Finally, the system was rinsed with 500 μL of 10 mM Tris-HCl solution.

## 3. Results

### 3.1. Proof of Principle

**Figure 4.**
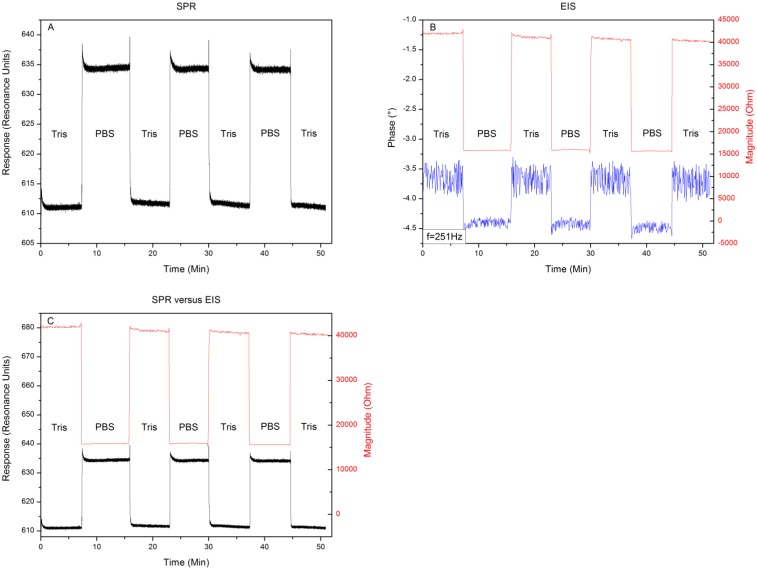
Alternating the media 10 mM Tris-HCl and 1× PBS buffer for three consecutive runs. (**A**) Shows the minimum shift when exchanging the media. (**B**) Illustrates the changes in amplitude and its phase at a frequency of 251 Hz, when exchanging the media. Comparing the signals, when plotted in the same graph, reveals that the read-out systems detected the media exchange simultaneously, without influencing each other (**C**).

Figure 4 shows the results when exchanging the media 10 mM Tris-HCl and 1× PBS buffer for three consecutive runs. The response to an exchange of buffer media can be seen in Figure 4A. Filling the reaction chamber with 10 mM Tris-HCl provides a local minimum of 610 ± 2 RU. Replacing this solution with 1× PBS buffer provides a local minimum in the intensity of reflected light of 635 ± 2 resonance units (RU). When repeating this medium exchange it can be seen that this result is highly reproducible with minor signs of drift. Figure 4B shows the changes in impedance and its phase at a frequency of 251 Hz, while exchanging the media. The 10 mM Tris-HCl solution provides a complex resistance of 40 kΩ ± 58 Ω and an accompanying phase of −3.7 ± 0.4°. When exchanging the 10 mM Tris-HCl solution with 1× PBS, the impedance decreases to 15.5 kΩ ± 35 Ω with a phase shift towards −4.4° ± 0.1°. Repeating the medium exchange for three consecutive runs shows again a high reproducibility of the values mentioned above with little drift or increasing error on the signal. To assess whether the EIS and SPR unit detected the media exchange at the same time, the minimum shift from Figure 4A and changes in impedance, depicted in Figure 4B are plotted in one graph as can be seen in Figure 4C. Comparing the signals, when plotted together in the same graph, reveals that the units detected the media exchange at the same time, without any signs of crosstalk or influencing each other. The results of the proof-of-principle test, described in Section 3.1 reveal that, when changing the medium from 10 mM Tris-HCl to 1× PBS and then back to 10 mM Tris-HCl, little shift in response or impedance occurs when comparing the 10 mM Tris-HCl plateaus. When comparing the effect sizes, very low noise levels can be observed in the SPR and EIS magnitude data. However the EIS phase data appears to be noisy. Exchanging media provides a predominantly resistive effect, which explains the minor change in phase (±1°). Having an error of ± 0.5° will therefore lead to a high noise level when compared to the small effect size.

### 3.2. Usability Test

**Figure 5.**
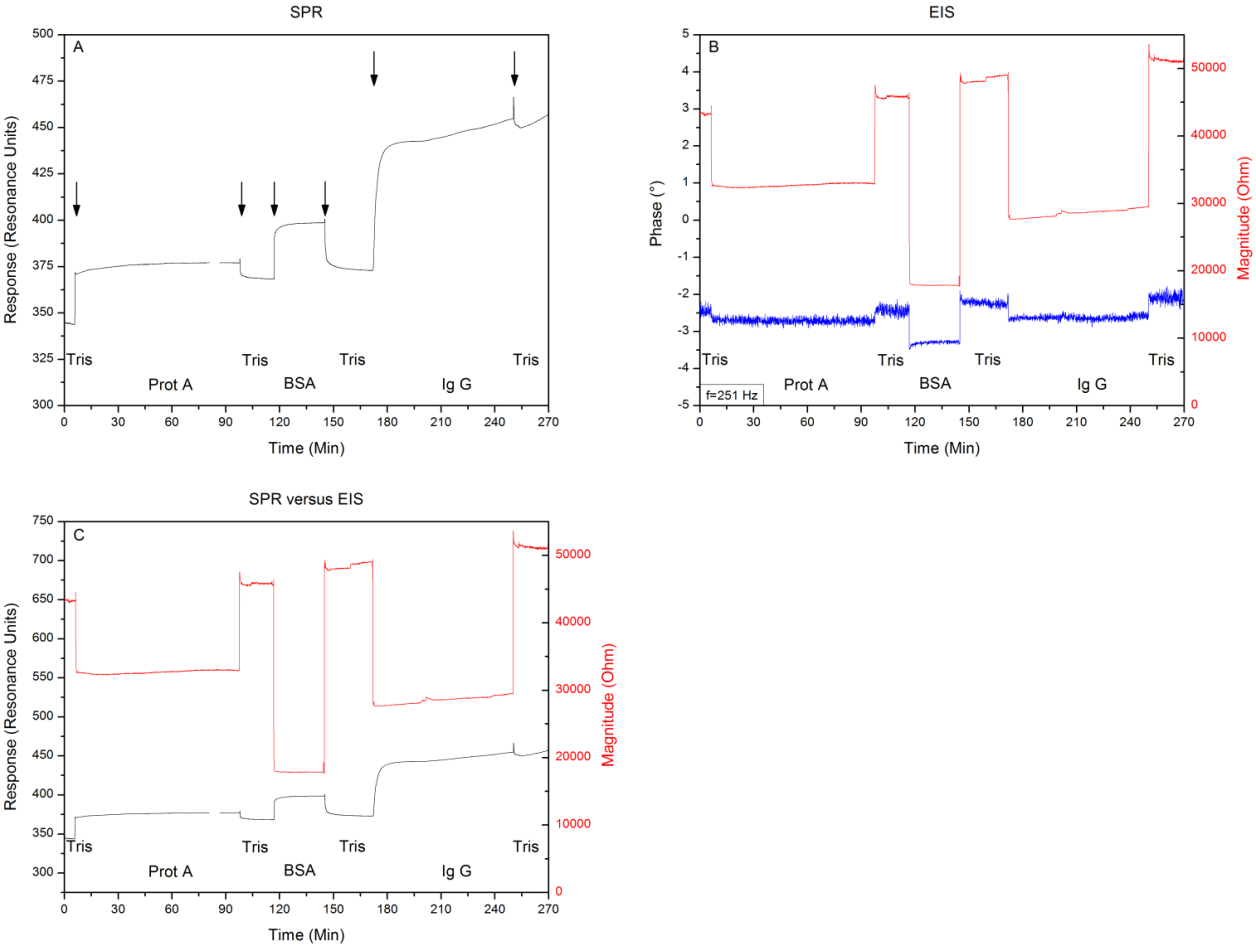
(**A**) Shows the minimum shift and (**B**) illustrates the changes in impedance and its phase at a frequency of 251 Hz, when attaching Protein A to the surface, blocking the non-occupied zone with BSA molecules and binding the IgG1 to the Protein A. Comparing the signals, when plotted in the same graph, reveals that the read-out systems detected each addition simultaneously (**C**).

After proving the system to be free of crosstalk and systematic errors, Topas® chips were functionalized with adsorbed Protein A as receptor for IgG1, then BSA blocker molecules were added and finally the binding of IgG1 was monitored simultaneously with SPR and EIS. Between each addition the system was rinsed with 500 μL of 10 mM Tris-HCl solution. Comparing the 10 mM Tris-HCl plateaus between additions provides the net effect of each addition and excludes any medium effects. Figure 5 shows the minimum shift and impedance throughout the entire experiment, arrows indicate the exchange of medium. Filling the reaction chamber with 10 mM Tris-HCl provides a local minimum of 340 ± 2 RU and an impedimetric value of 43 kΩ ± 58 Ω. Then Protein A is added as a receptor for the IgG1 antibodies. The system is then rinsed for the first time with 10 mM Tris-HCl, providing a minimum of 370 ± 2 RU and an impedance of 46 kΩ ± 41 Ω.

After the BSA solution is added, the system is rinsed for a second time with 10 mM Tris-HCl, leading to a local minimum of 375 ± 2 RU and an impedance of 48 kΩ ± 53 Ω. Finally the IgG1 solution is pumped into the reaction chamber, followed by a last washing step of 10 mM Tris-HCl solution, which gives a local minimum of 460 ± 2 RU and an impedimetric value of 51 kΩ ± 49 Ω. Comparing the signals, by plotting them in the same graph (Figure 5C), reveals simultaneous detections without crosstalk.

## 4. Discussion and Conclusions

**Figure 6.**
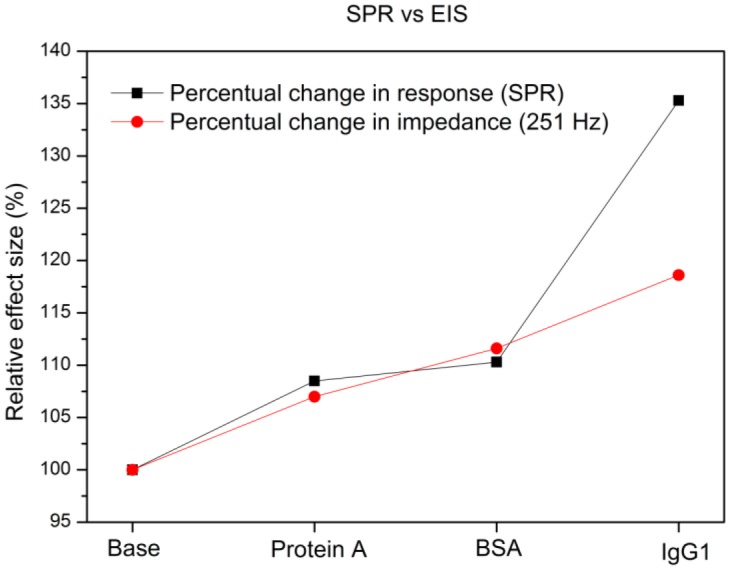
Relative effect sizes for EIS and SPR for each addition during the usability test. The effect sizes are normalized to their initial value and appear to be in the same order of magnitude.

In this study we explored the possibility of combining two different read-out techniques (EIS and SPR). After establishing a combined setup (Figure 2), a proof of principle experiment was conducted. 10 mM Tris-HCl and 1× PBS buffer was introduced in an alternating manner to the reaction chamber of the PDMS flow-cell. The results have shown that these media changes could be detected both by EIS and SPR and when changing the medium from 10 mM Tris-HCl to 1× PBS and then back to 10 mM Tris-HCl, no shift in minimum or impedance occurs. Plotting the results in one graph (Figure 4C) revealed that EIS and SPR detected the changes simultaneously, without any evidence of crosstalk. After the successful proof of principle experiment a usability test was conducted with Protein A as receptors and IgG1 as target molecules. The possibility to detect the binding of IgG1 to Protein A simultaneously by EIS and SPR was investigated. Comparing the 10 mM Tris-HCl plateaus between additions provided the net effect of each addition and excluded any media effects. Figure 6 illustrates the percentual effect sizes for EIS and SPR for each addition.

The effect sizes have been normalized to their initial value (reaction chamber filled with 10 mM Tris-HCl). Adding Protein A to the reaction chamber resulted in a minimum shift of 8.5% and an increase in impedance by 7.0%. Blocking the non-occupied zone with the BSA molecules resulted in a minimum shift of 10.3% and an increase in impedance of 11.6%. The final attachment of IgG1 to Protein A led to a minimum shift of 35.3% and an increase in impedance of 18.6%. Overall error on the results shown in Section 4, lie in the order of ±0.1%. Despite the fact these results originate from two independent physical phenomena, the percentual minimum shifts and changes in impedance are on the same order of magnitude. However, for the attachment of IgG1, a distinct difference in effect size obtained by the two technologies can be observed. This could be explained through the fact that SPR is sensitive to changes in dielectric constant, whereas impedance is more sensitive to changes in resistance and charge distribution. Attaching IgG1 appears to create a greater change in dielectric constant as compared to the change in resistance or charge distribution. Additionally, the structure of the set-up may also contribute to this phenomenon: the dielectric constant is measured in parallel to the surface. As IgG1 covers almost the entire surface, it will have a great and distinct effect on the dielectric constant. Impedance is measured top-down. This means that covering an entire surface does not imply an excessive effect size, because the resistive layer which is added is relatively thin. In this case the effect size measured vertically is smaller than the effect size measured horizontally. The results indicate that a merge between SPR and EIS is technically possible. Combining these technologies into one read-out system provides a real time reference for two different physical phenomena and facilitates the interpretation of data.
